# A Large Language Model Leaderboard for Clinical Note Entity Extraction

**DOI:** 10.23889/ijpds.v11i5.3811

**Published:** 2026-07-06

**Authors:** Elliot Martin, Seungwon Lee, Kiarash Riazi, Cathy Eastwood, Hude Quan

**Affiliations:** 1 Provincial Research Data Services, Health Shared Services, Calgary, Canada; 2 Centre for Health Informatics, University of Calgary, Calgary, Canada; 3 Department of Community Health Sciences, University of Calgary, Calgary, Canada; 4 Libin Cardiovascular Institute, University of Calgary, Calgary, Canada

## Objective

Large language models (LLMs) have the potential to revolutionize how population-level health research is conducted by automatically abstracting data that would otherwise be unavailable. However, few results are available on real clinical notes. We developed an LLM leaderboard showing how open-source LLMs perform at entity extraction on unseen clinical notes.

## Approach

EMR data, including free-text notes, were linked to a chart-review cohort comprising 10,659 adults admitted to a hospital in Calgary, Canada, between 2017 and 2022, with data on comorbidities. We then attempted to replicate this chart review with multiple open-source LLMs in a secure computing environment. Chart review results served as the reference standard.

## Results

There was a wide variation in performance among the examined LLMs: the smallest, Llama 3.2 3B, had a high mean sensitivity of 0.97 but low PPV at 0.4; the largest, Llama-3-70B, showed a high mean sensitivity of 0.96 and greatly improved PPV of 0.7; in-between these in size, phi 4 demonstrated a more balanced performance with a mean sensitivity of 0.81 and PPV of 0.77. However, the results varied considerably across conditions, with quirks specific to each model.

## Conclusions

LLMs are already available that can perform entity extraction well enough to be considered in place of some administrative data. With rapid developments in the field, a leaderboard based on real clinical data is vital for informing researchers on best practices for integrating the latest AI techniques into their data practices.

